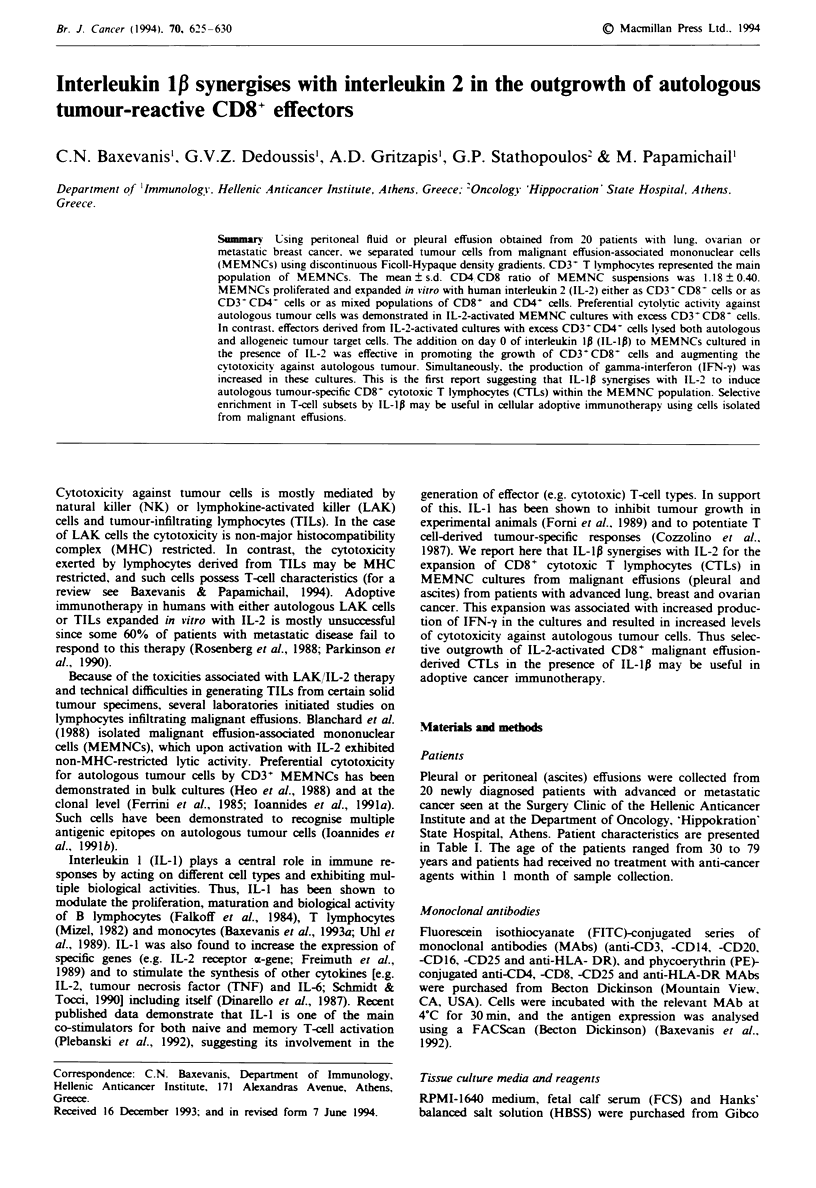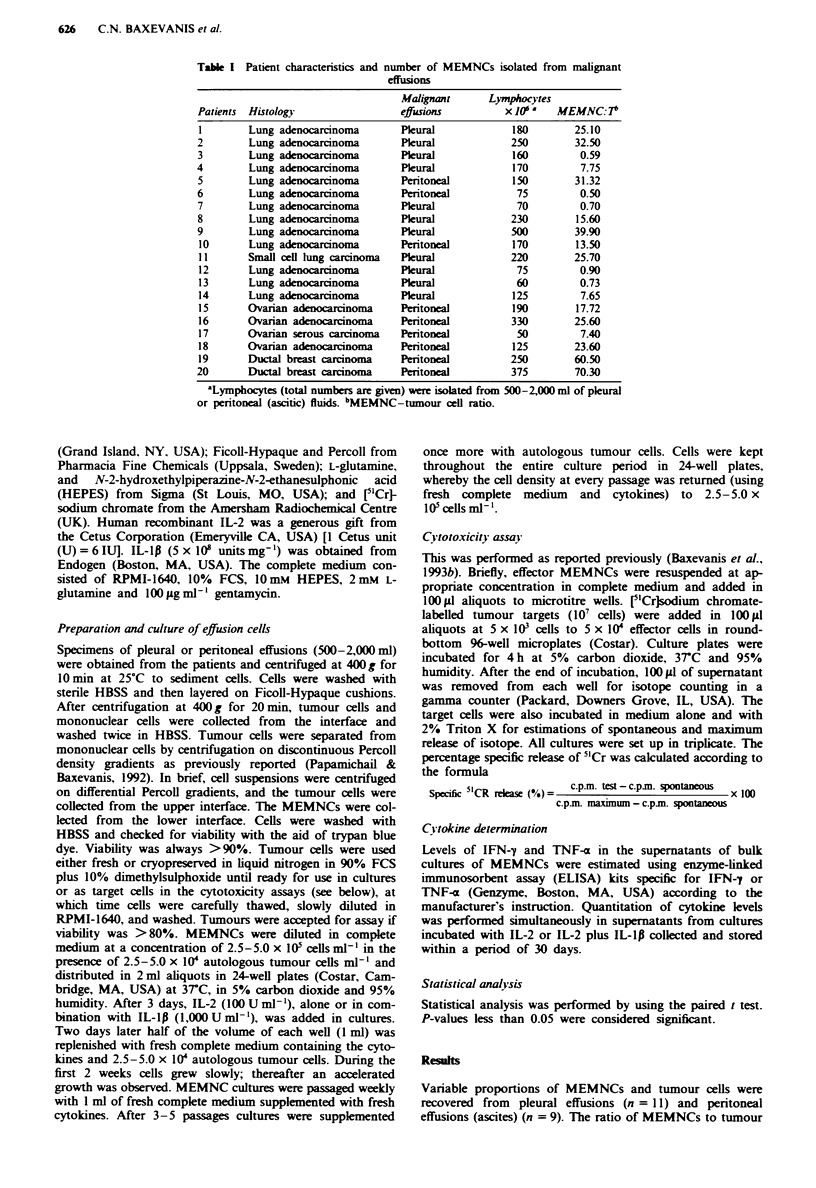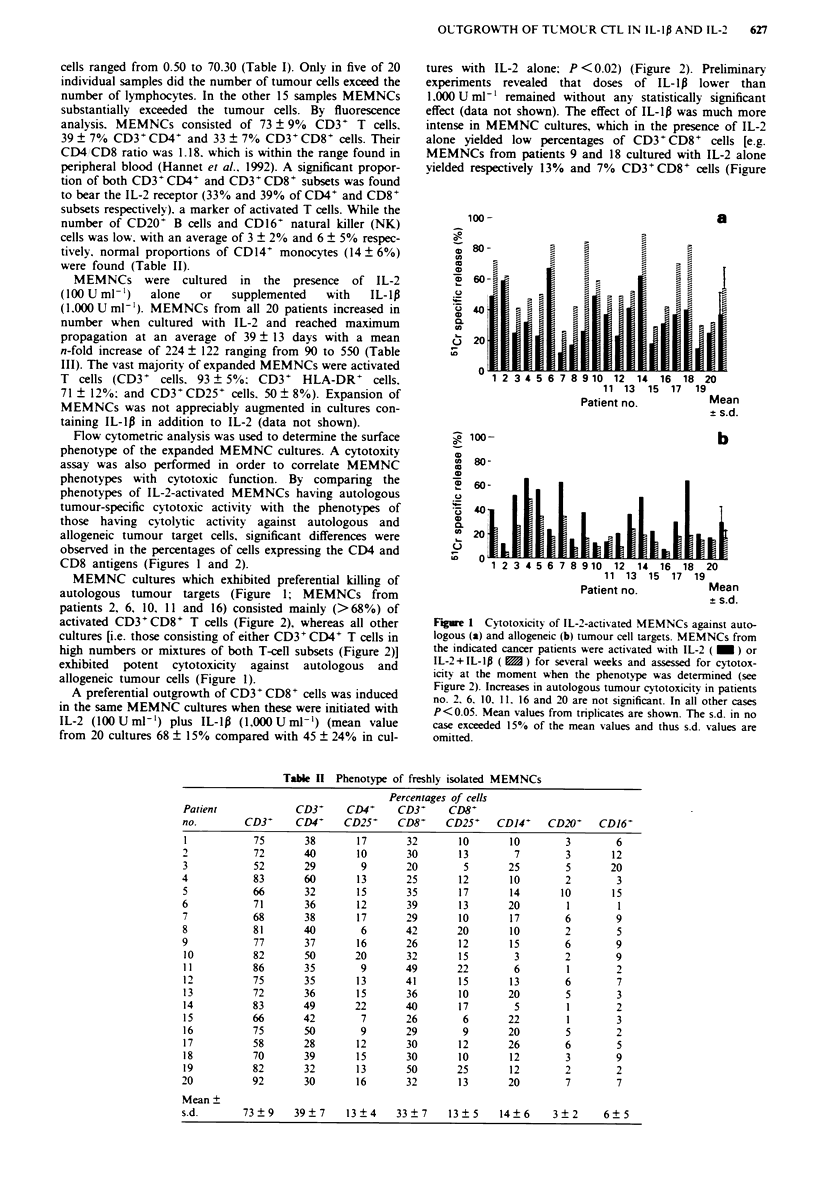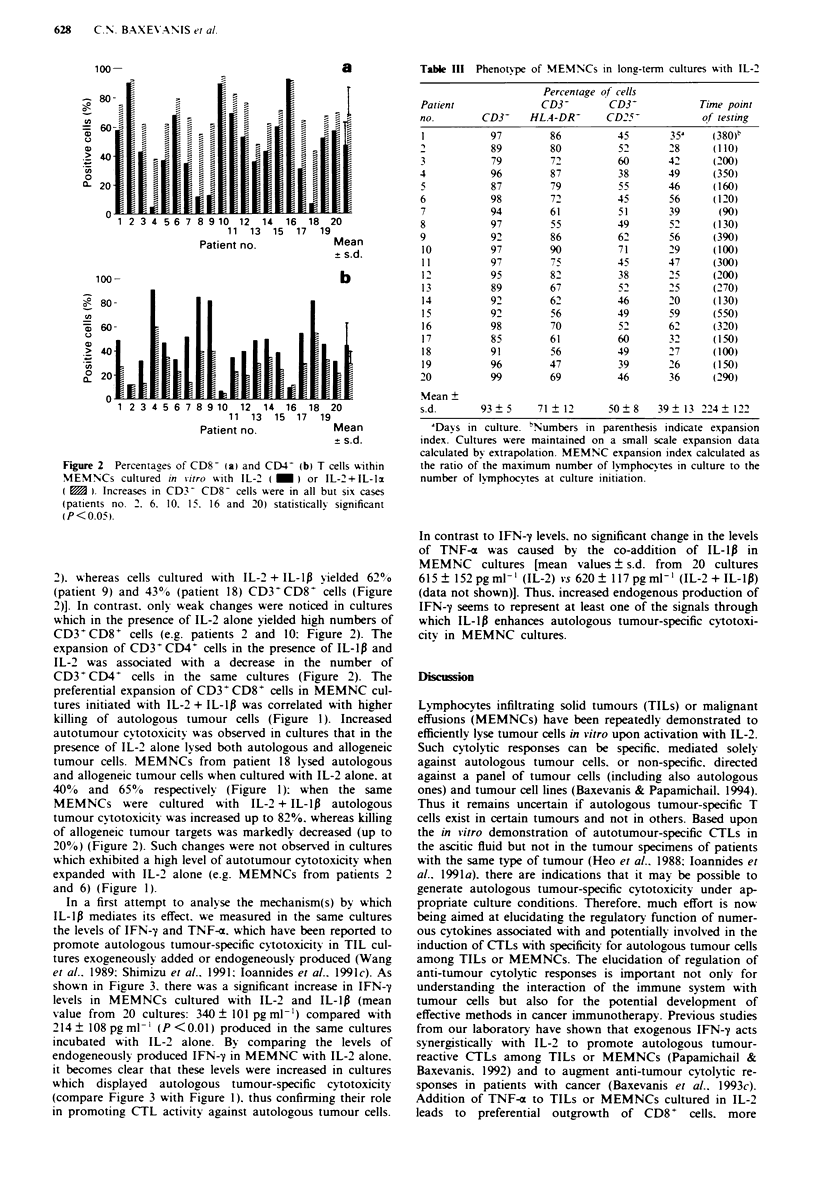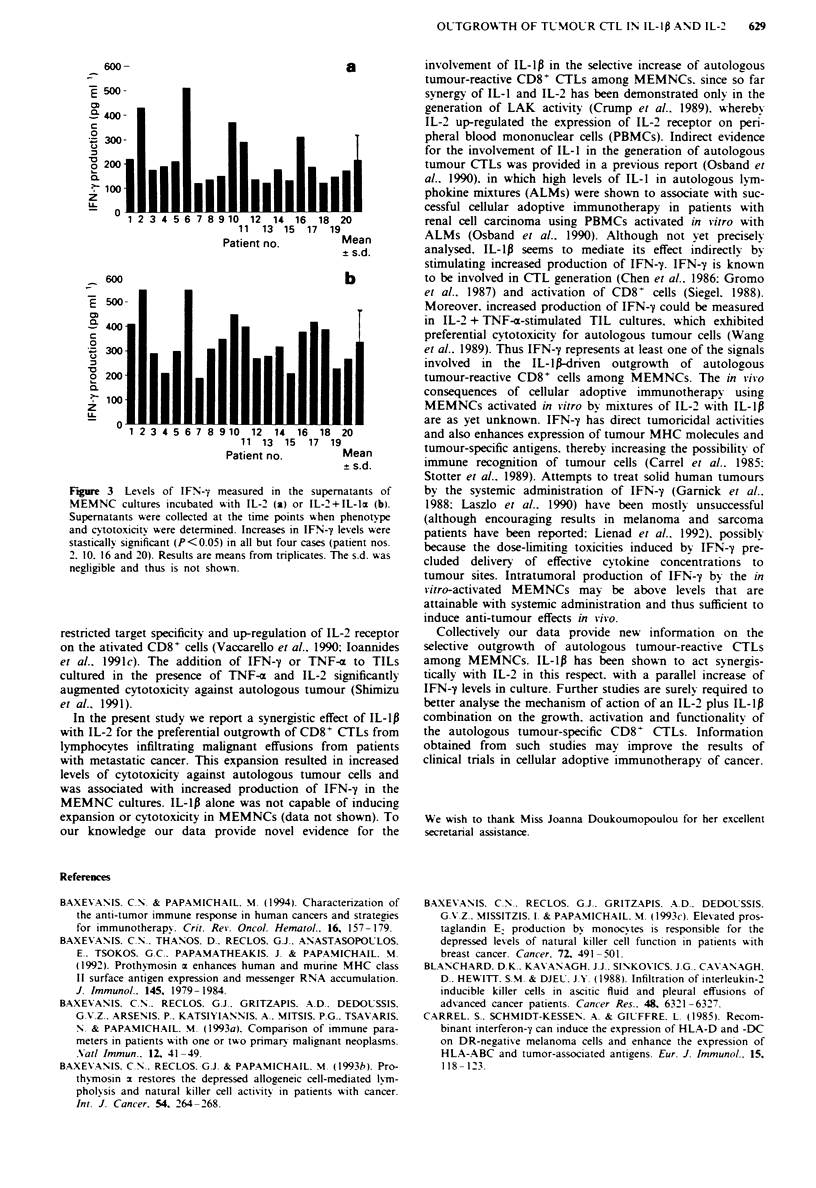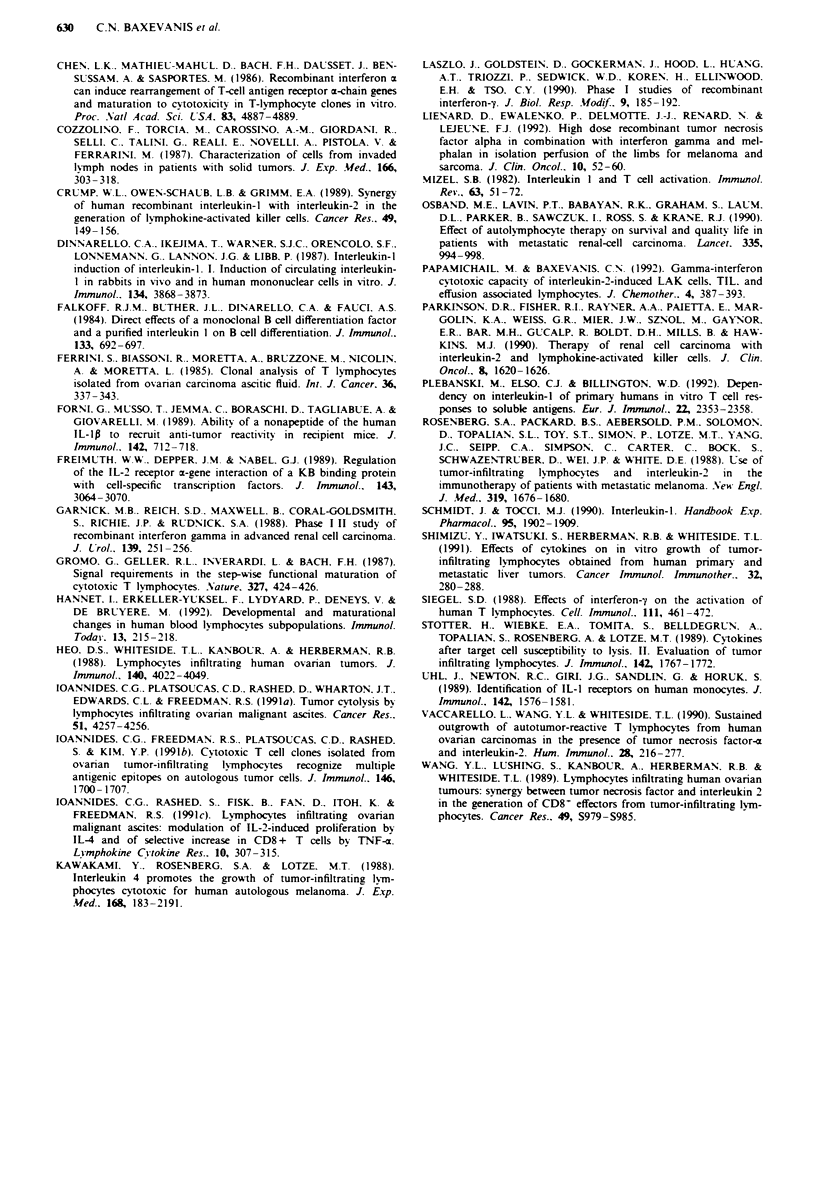# Interleukin 1 beta synergises with interleukin 2 in the outgrowth of autologous tumour-reactive CD8+ effectors.

**DOI:** 10.1038/bjc.1994.361

**Published:** 1994-10

**Authors:** C. N. Baxevanis, G. V. Dedoussis, A. D. Gritzapis, G. P. Stathopoulos, M. Papamichail

**Affiliations:** Department of Immunology, Hellenic Anticancer Institute, Athens, Greece.

## Abstract

Using peritoneal fluid or pleural effusion obtained from 20 patients with lung, ovarian or metastatic breast cancer, we separated tumour cells from malignant effusion-associated mononuclear cells (MEMNCs) using discontinuous Ficoll-Hypaque density gradients. CD3+ T lymphocytes represented the main population of MEMNCs. The mean +/- s.d. CD4/CD8 ratio of MEMNC suspensions was 1.18 +/- 0.40. MEMNCs proliferated and expanded in vitro with human interleukin 2 (IL-2) either as CD3+ CD8+ cells or as CD3+ CD4+ cells or as mixed populations of CD8+ and CD4+ cells. Preferential cytolytic activity against autologous tumour cells was demonstrated in IL-2-activated MEMNC cultures with excess CD3+ CD8+ cells. In contrast, effectors derived from IL-2-activated cultures with excess CD3+ CD4+ cells lysed both autologous and allogeneic tumour target cells. The addition on day 0 of interleukin 1 beta (IL-1 beta) to MEMNCs cultured in the presence of IL-2 was effective in promoting the growth of CD3+ CD8+ cells and augmenting the cytotoxicity against autologous tumour. Simultaneously, the production of gamma-interferon (IFN-gamma) was increased in these cultures. This is the first report suggesting that IL-1 beta synergises with IL-2 to induce autologous tumour-specific CD8+ cytotoxic T lymphocytes (CTLs) within the MEMNC population. Selective enrichment in T-cell subsets by IL-1 beta may be useful in cellular adoptive immunotherapy using cells isolated from malignant effusions.


Br. J. Cancer (1994), 70, 625–630 © Macmillan Press Ltd., 1994

### Interleukin 1β synergises with interleukin 2 in the outgrowth of autologous tumour-reactive CD8+ effectors

C.N. Baxevanis[1], G.V.Z. Dedoussis[1], A.D. Gritzapis[1], G.P. Stathopoulos[2] & M. Papamichail[1]

*Department of [1]Immunology, Hellenic Anticancer Institute, Athens, Greece; [2]Oncology 'Hippocration' State Hospital, Athens, Greece.*

**Summary** Using peritoneal fluid or pleural effusion obtained from 20 patients with lung, ovarian or metastatic breast cancer, we separated tumour cells from malignant effusion-associated mononuclear cells (MEMNCs) using discontinuous Ficoll-Hypaque density gradients. $CD3^+$ T lymphocytes represented the main population of MEMNCs. The mean ± s.d. CD4/CD8 ratio of MEMNC suspensions was 1.18 ± 0.40. MEMNCs proliferated and expanded *in vitro* with human interleukin 2 (IL-2) either as $CD3^+CD8^+$ cells or as $CD3^+CD4^+$ cells or as mixed populations of $CD8^+$ and $CD4^+$ cells. Preferential cytolytic activity against autologous tumour cells was demonstrated in IL-2-activated MEMNC cultures with excess $CD3^+CD8^+$ cells. In contrast, effectors derived from IL-2-activated cultures with excess $CD3^+CD4^+$ cells lysed both autologous and allogeneic tumour target cells. The addition on day 0 of interleukin 1β (IL-1β) to MEMNCs cultured in the presence of IL-2 was effective in promoting the growth of $CD3^+CD8^+$ cells and augmenting the cytotoxicity against autologous tumour. Simultaneously, the production of gamma-interferon (IFN-γ) was increased in these cultures. This is the first report suggesting that IL-1β synergises with IL-2 to induce autologous tumour-specific $CD8^+$ cytotoxic T lymphocytes (CTLs) within the MEMNC population. Selective enrichment in T-cell subsets by IL-1β may be useful in cellular adoptive immunotherapy using cells isolated from malignant effusions.

Cytotoxicity against tumour cells is mostly mediated by natural killer (NK) or lymphokine-activated killer (LAK) cells and tumour-infiltrating lymphocytes (TILs). In the case of LAK cells the cytotoxicity is non-major histocompatibility complex (MHC) restricted. In contrast, the cytotoxicity exerted by lymphocytes derived from TILs may be MHC restricted, and such cells possess T-cell characteristics (for a review see Baxevanis & Papamichail, 1994). Adoptive immunotherapy in humans with either autologous LAK cells or TILs expanded *in vitro* with IL-2 is mostly unsuccessful since some 60% of patients with metastatic disease fail to respond to this therapy (Rosenberg *et al*., 1988; Parkinson *et al*., 1990).

Because of the toxicities associated with LAK/IL-2 therapy and technical difficulties in generating TILs from certain solid tumour specimens, several laboratories initiated studies on lymphocytes infiltrating malignant effusions. Blanchard *et al.* (1988) isolated malignant effusion-associated mononuclear cells (MEMNCs), which upon activation with IL-2 exhibited non-MHC-restricted lytic activity. Preferential cytotoxicity for autologous tumour cells by $CD3^+$ MEMNCs has been demonstrated in bulk cultures (Heo *et al*., 1988) and at the clonal level (Ferrini *et al*., 1985; Ioannides *et al*., 1991*a*). Such cells have been demonstrated to recognise multiple antigenic epitopes on autologous tumour cells (Ioannides *et al*., 1991*b*).

Interleukin 1 (IL-1) plays a central role in immune responses by acting on different cell types and exhibiting multiple biological activities. Thus, IL-1 has been shown to modulate the proliferation, maturation and biological activity of B lymphocytes (Falkoff *et al*., 1984), T lymphocytes (Mizel, 1982) and monocytes (Baxevanis *et al*., 1993*a*; Uhl *et al*., 1989). IL-1 was also found to increase the expression of specific genes (e.g. IL-2 receptor α-gene; Freimuth *et al*., 1989) and to stimulate the synthesis of other cytokines [e.g. IL-2, tumour necrosis factor (TNF) and IL-6; Schmidt & Tocci, 1990] including itself (Dinarello *et al*., 1987). Recent published data demonstrate that IL-1 is one of the main co-stimulators for both naive and memory T-cell activation (Plebanski *et al*., 1992), suggesting its involvement in the generation of effector (e.g. cytotoxic) T-cell types. In support of this, IL-1 has been shown to inhibit tumour growth in experimental animals (Forni *et al*., 1989) and to potentiate T cell-derived tumour-specific responses (Cozzolino *et al*., 1987). We report here that IL-1β synergises with IL-2 for the expansion of $CD8^+$ cytotoxic T lymphocytes (CTLs) in MEMNC cultures from malignant effusions (pleural and ascites) from patients with advanced lung, breast and ovarian cancer. This expansion was associated with increased production of IFN-γ in the cultures and resulted in increased levels of cytotoxicity against autologous tumour cells. Thus selective outgrowth of IL-2-activated $CD8^+$ malignant effusion-derived CTLs in the presence of IL-1β may be useful in adoptive cancer immunotherapy.

### Materials and methods

### *Patients*

Pleural or peritoneal (ascites) effusions were collected from 20 newly diagnosed patients with advanced or metastatic cancer seen at the Surgery Clinic of the Hellenic Anticancer Institute and at the Department of Oncology, 'Hippokration' State Hospital, Athens. Patient characteristics are presented in Table I. The age of the patients ranged from 30 to 79 years and patients had received no treatment with anti-cancer agents within 1 month of sample collection.

### *Monoclonal antibodies*

Fluorescein isothiocyanate (FITC)-conjugated series of monoclonal antibodies (MAbs) (anti-CD3, -CD14, -CD20, -CD16, -CD25 and anti-HLA- DR), and phycoerythrin (PE)-conjugated anti-CD4, -CD8, -CD25 and anti-HLA-DR MAbs were purchased from Becton Dickinson (Mountain View, CA, USA). Cells were incubated with the relevant MAb at 4°C for 30 min, and the antigen expression was analysed using a FACScan (Becton Dickinson) (Baxevanis *et al*., 1992).

### *Tissue culture media and reagents*

RPMI-1640 medium, fetal calf serum (FCS) and Hanks' balanced salt solution (HBSS) were purchased from Gibco

Correspondence: C.N. Baxevanis, Department of Immunology, Hellenic Anticancer Institute, 171 Alexandras Avenue, Athens, Greece.
Received 16 December 1993; and in revised form 7 June 1994.

Table I Patient characteristics and number of MEMNCs isolated from malignant effusions

| Patients | Histology | Malignant effusions | Lymphocytes $\times 10^6$ a | MEMNC:T b |
|---|---|---|---|---|
| 1 | Lung adenocarcinoma | Pleural | 180 | 25.10 |
| 2 | Lung adenocarcinoma | Pleural | 250 | 32.50 |
| 3 | Lung adenocarcinoma | Pleural | 160 | 0.59 |
| 4 | Lung adenocarcinoma | Pleural | 170 | 7.75 |
| 5 | Lung adenocarcinoma | Peritoneal | 150 | 31.32 |
| 6 | Lung adenocarcinoma | Peritoneal | 75 | 0.50 |
| 7 | Lung adenocarcinoma | Pleural | 70 | 0.70 |
| 8 | Lung adenocarcinoma | Pleural | 230 | 15.60 |
| 9 | Lung adenocarcinoma | Pleural | 500 | 39.90 |
| 10 | Lung adenocarcinoma | Peritoneal | 170 | 13.50 |
| 11 | Small cell lung carcinoma | Pleural | 220 | 25.70 |
| 12 | Lung adenocarcinoma | Pleural | 75 | 0.90 |
| 13 | Lung adenocarcinoma | Pleural | 60 | 0.73 |
| 14 | Lung adenocarcinoma | Pleural | 125 | 7.65 |
| 15 | Ovarian adenocarcinoma | Peritoneal | 190 | 17.72 |
| 16 | Ovarian adenocarcinoma | Peritoneal | 330 | 25.60 |
| 17 | Ovarian serous carcinoma | Peritoneal | 50 | 7.40 |
| 18 | Ovarian adenocarcinoma | Peritoneal | 125 | 23.60 |
| 19 | Ductal breast carcinoma | Peritoneal | 250 | 60.50 |
| 20 | Ductal breast carcinoma | Peritoneal | 375 | 70.30 |

aLymphocytes (total numbers are given) were isolated from 500–2,000 ml of pleural or peritoneal (ascitic) fluids. bMEMNC–tumour cell ratio.

(Grand Island, NY, USA); Ficoll-Hypaque and Percoll from Pharmacia Fine Chemicals (Uppsala, Sweden); L-glutamine, and N-2-hydroxethylpiperazine-N-2-ethanesulphonic acid (HEPES) from Sigma (St Louis, MO, USA); and [$^{51}$Cr]-sodium chromate from the Amersham Radiochemical Centre (UK). Human recombinant IL-2 was a generous gift from the Cetus Corporation (Emeryville CA, USA) [1 Cetus unit (U) = 6 IU]. IL-1$\beta$ ($5 \times 10^8$ units $mg^{-1}$) was obtained from Endogen (Boston, MA, USA). The complete medium consisted of RPMI-1640, 10% FCS, 10 mM HEPES, 2 mM L-glutamine and 100 $\mu g\, ml^{-1}$ gentamycin.

*Preparation and culture of effusion cells*

Specimens of pleural or peritoneal effusions (500–2,000 ml) were obtained from the patients and centrifuged at 400 *g* for 10 min at 25°C to sediment cells. Cells were washed with sterile HBSS and then layered on Ficoll-Hypaque cushions. After centrifugation at 400 *g* for 20 min, tumour cells and mononuclear cells were collected from the interface and washed twice in HBSS. Tumour cells were separated from mononuclear cells by centrifugation on discontinuous Percoll density gradients as previously reported (Papamichail & Baxevanis, 1992). In brief, cell suspensions were centrifuged on differential Percoll gradients, and the tumour cells were collected from the upper interface. The MEMNCs were collected from the lower interface. Cells were washed with HBSS and checked for viability with the aid of trypan blue dye. Viability was always >90%. Tumour cells were used either fresh or cryopreserved in liquid nitrogen in 90% FCS plus 10% dimethylsulphoxide until ready for use in cultures or as target cells in the cytotoxicity assays (see below), at which time cells were carefully thawed, slowly diluted in RPMI-1640, and washed. Tumours were accepted for assay if viability was >80%. MEMNCs were diluted in complete medium at a concentration of $2.5-5.0 \times 10^5$ cells $ml^{-1}$ in the presence of $2.5-5.0 \times 10^4$ autologous tumour cells $ml^{-1}$ and distributed in 2 ml aliquots in 24-well plates (Costar, Cambridge, MA, USA) at 37°C, in 5% carbon dioxide and 95% humidity. After 3 days, IL-2 (100 U $ml^{-1}$), alone or in combination with IL-1$\beta$ (1,000 U $ml^{-1}$), was added in cultures. Two days later half of the volume of each well (1 ml) was replenished with fresh complete medium containing the cytokines and $2.5-5.0 \times 10^4$ autologous tumour cells. During the first 2 weeks cells grew slowly; thereafter an accelerated growth was observed. MEMNC cultures were passaged weekly with 1 ml of fresh complete medium supplemented with fresh cytokines. After 3–5 passages cultures were supplemented once more with autologous tumour cells. Cells were kept throughout the entire culture period in 24-well plates, whereby the cell density at every passage was returned (using fresh complete medium and cytokines) to $2.5-5.0 \times 10^5$ cells $ml^{-1}$.

*Cytotoxicity assay*

This was performed as reported previously (Baxevanis *et al.*, 1993*b*). Briefly, effector MEMNCs were resuspended at appropriate concentration in complete medium and added in 100 µl aliquots to microtitre wells. [$^{51}$Cr]sodium chromate-labelled tumour targets ($10^7$ cells) were added in 100 µl aliquots at $5 \times 10^3$ cells to $5 \times 10^4$ effector cells in round-bottom 96-well microplates (Costar). Culture plates were incubated for 4 h at 5% carbon dioxide, 37°C and 95% humidity. After the end of incubation, 100 µl of supernatant was removed from each well for isotope counting in a gamma counter (Packard, Downers Grove, IL, USA). The target cells were also incubated in medium alone and with 2% Triton X for estimations of spontaneous and maximum release of isotope. All cultures were set up in triplicate. The percentage specific release of $^{51}$Cr was calculated according to the formula

$$\text{Specific } ^{51}\text{CR release } (\%) = \frac{\text{c.p.m. test} - \text{c.p.m. spontaneous}}{\text{c.p.m. maximum} - \text{c.p.m. spontaneous}} \times 100$$

*Cytokine determination*

Levels of IFN-$\gamma$ and TNF-$\alpha$ in the supernatants of bulk cultures of MEMNCs were estimated using enzyme-linked immunosorbent assay (ELISA) kits specific for IFN-$\gamma$ or TNF-$\alpha$ (Genzyme, Boston, MA, USA) according to the manufacturer's instruction. Quantitation of cytokine levels was performed simultaneously in supernatants from cultures incubated with IL-2 or IL-2 plus IL-1$\beta$ collected and stored within a period of 30 days.

*Statistical analysis*

Statistical analysis was performed by using the paired *t* test. *P*-values less than 0.05 were considered significant.

### Results

Variable proportions of MEMNCs and tumour cells were recovered from pleural effusions ($n = 11$) and peritoneal effusions (ascites) ($n = 9$). The ratio of MEMNCs to tumour cells ranged from 0.50 to 70.30 (Table I). Only in five of 20 individual samples did the number of tumour cells exceed the number of lymphocytes. In the other 15 samples MEMNCs substantially exceeded the tumour cells. By fluorescence analysis, MEMNCs consisted of 73 ± 9% $CD3^+$ T cells, 39 ± 7% $CD3^+CD4^+$ and 33 ± 7% $CD3^+CD8^+$ cells. Their CD4/CD8 ratio was 1.18, which is within the range found in peripheral blood (Hannet *et al.*, 1992). A significant proportion of both $CD3^+CD4^+$ and $CD3^+CD8^+$ subsets was found to bear the IL-2 receptor (33% and 39% of $CD4^+$ and $CD8^+$ subsets respectively), a marker of activated T cells. While the number of $CD20^+$ B cells and $CD16^+$ natural killer (NK) cells was low, with an average of 3 ± 2% and 6 ± 5% respectively, normal proportions of $CD14^+$ monocytes (14 ± 6%) were found (Table II).

MEMNCs were cultured in the presence of IL-2 (100 U $ml^{-1}$) alone or supplemented with IL-1β (1,000 U $ml^{-1}$). MEMNCs from all 20 patients increased in number when cultured with IL-2 and reached maximum propagation at an average of 39 ± 13 days with a mean $n$-fold increase of 224 ± 122 ranging from 90 to 550 (Table III). The vast majority of expanded MEMNCs were activated T cells ($CD3^+$ cells, 93 ± 5%; $CD3^+$ $HLA\text{-}DR^+$ cells, 71 ± 12%; and $CD3^+CD25^+$ cells, 50 ± 8%). Expansion of MEMNCs was not appreciably augmented in cultures containing IL-1β in addition to IL-2 (data not shown).

Flow cytometric analysis was used to determine the surface phenotype of the expanded MEMNC cultures. A cytotoxity assay was also performed in order to correlate MEMNC phenotypes with cytotoxic function. By comparing the phenotypes of IL-2-activated MEMNCs having autologous tumour-specific cytotoxic activity with the phenotypes of those having cytolytic activity against autologous and allogeneic tumour target cells, significant differences were observed in the percentages of cells expressing the CD4 and CD8 antigens (Figures 1 and 2).

MEMNC cultures which exhibited preferential killing of autologous tumour targets (Figure 1; MEMNCs from patients 2, 6, 10, 11 and 16) consisted mainly (>68%) of activated $CD3^+CD8^+$ T cells (Figure 2), whereas all other cultures [i.e. those consisting of either $CD3^+CD4^+$ T cells in high numbers or mixtures of both T-cell subsets (Figure 2)] exhibited potent cytotoxicity against autologous and allogeneic tumour cells (Figure 1).

A preferential outgrowth of $CD3^+CD8^+$ cells was induced in the same MEMNC cultures when these were initiated with IL-2 (100 U $ml^{-1}$) plus IL-1β (1,000 U $ml^{-1}$) (mean value from 20 cultures 68 ± 15% compared with 45 ± 24% in cultures with IL-2 alone; $P < 0.02$) (Figure 2). Preliminary experiments revealed that doses of IL-1β lower than 1,000 U $ml^{-1}$ remained without any statistically significant effect (data not shown). The effect of IL-1β was much more intense in MEMNC cultures, which in the presence of IL-2 alone yielded low percentages of $CD3^+CD8^+$ cells [e.g. MEMNCs from patients 9 and 18 cultured with IL-2 alone yielded respectively 13% and 7% $CD3^+CD8^+$ cells (Figure

**Figure 1** Cytotoxicity of IL-2-activated MEMNCs against autologous (**a**) and allogeneic (**b**) tumour cell targets. MEMNCs from the indicated cancer patients were activated with IL-2 (■) or IL-2 + IL-1β (▨) for several weeks and assessed for cytotoxicity at the moment when the phenotype was determined (see Figure 2). Increases in autologous tumour cytotoxicity in patients no. 2, 6, 10, 11, 16 and 20 are not significant. In all other cases $P < 0.05$. Mean values from triplicates are shown. The s.d. in no case exceeded 15% of the mean values and thus s.d. values are omitted.

**Table II** Phenotype of freshly isolated MEMNCs

| Patient no. | $CD3^+$ | $CD3^+$ $CD4^+$ | $CD4^+$ $CD25^+$ | $CD3^+$ $CD8^+$ | $CD8^+$ $CD25^+$ | $CD14^+$ | $CD20^+$ | $CD16^+$ |
|---|---|---|---|---|---|---|---|---|
| | *Percentages of cells* | | | | | | | |
| 1 | 75 | 38 | 17 | 32 | 10 | 10 | 3 | 6 |
| 2 | 72 | 40 | 10 | 30 | 13 | 7 | 3 | 12 |
| 3 | 52 | 29 | 9 | 20 | 5 | 25 | 5 | 20 |
| 4 | 83 | 60 | 13 | 25 | 12 | 10 | 2 | 3 |
| 5 | 66 | 32 | 15 | 35 | 17 | 14 | 10 | 15 |
| 6 | 71 | 36 | 12 | 39 | 13 | 20 | 1 | 1 |
| 7 | 68 | 38 | 17 | 29 | 10 | 17 | 6 | 9 |
| 8 | 81 | 40 | 6 | 42 | 20 | 10 | 2 | 5 |
| 9 | 77 | 37 | 16 | 26 | 12 | 15 | 6 | 9 |
| 10 | 82 | 50 | 20 | 32 | 15 | 3 | 2 | 9 |
| 11 | 86 | 35 | 9 | 49 | 22 | 6 | 1 | 2 |
| 12 | 75 | 35 | 13 | 41 | 15 | 13 | 6 | 7 |
| 13 | 72 | 36 | 15 | 36 | 10 | 20 | 5 | 3 |
| 14 | 83 | 49 | 22 | 40 | 17 | 5 | 1 | 2 |
| 15 | 66 | 42 | 7 | 26 | 6 | 22 | 1 | 3 |
| 16 | 75 | 50 | 9 | 29 | 9 | 20 | 5 | 2 |
| 17 | 58 | 28 | 12 | 30 | 12 | 26 | 6 | 5 |
| 18 | 70 | 39 | 15 | 30 | 10 | 12 | 3 | 9 |
| 19 | 82 | 32 | 13 | 50 | 25 | 12 | 2 | 2 |
| 20 | 92 | 30 | 16 | 32 | 13 | 20 | 7 | 7 |
| Mean ± s.d. | 73 ± 9 | 39 ± 7 | 13 ± 4 | 33 ± 7 | 13 ± 5 | 14 ± 6 | 3 ± 2 | 6 ± 5 |

**Figure 2** Percentages of CD8+ (**a**) and CD4+ (**b**) T cells within MEMNCs cultured *in vitro* with IL-2 ( ■ ) or IL-2 + IL-1α ( ▨ ). Increases in CD3+ CD8+ cells were in all but six cases (patients no. 2, 6, 10, 15, 16 and 20) statistically significant ($P < 0.05$).

2), whereas cells cultured with IL-2 + IL-1β yielded 62% (patient 9) and 43% (patient 18) CD3+CD8+ cells (Figure 2)]. In contrast, only weak changes were noticed in cultures which in the presence of IL-2 alone yielded high numbers of CD3+CD8+ cells (e.g. patients 2 and 10; Figure 2). The expansion of CD3+CD4+ cells in the presence of IL-1β and IL-2 was associated with a decrease in the number of CD3+CD4+ cells in the same cultures (Figure 2). The preferential expansion of CD3+CD8+ cells in MEMNC cultures initiated with IL-2 + IL-1β was correlated with higher killing of autologous tumour cells (Figure 1). Increased autotumour cytotoxicity was observed in cultures that in the presence of IL-2 alone lysed both autologous and allogeneic tumour cells. MEMNCs from patient 18 lysed autologous and allogeneic tumour cells when cultured with IL-2 alone, at 40% and 65% respectively (Figure 1); when the same MEMNCs were cultured with IL-2 + IL-1β autologous tumour cytotoxicity was increased up to 82%, whereas killing of allogeneic tumour targets was markedly decreased (up to 20%) (Figure 2). Such changes were not observed in cultures which exhibited a high level of autotumour cytotoxicity when expanded with IL-2 alone (e.g. MEMNCs from patients 2 and 6) (Figure 1).

In a first attempt to analyse the mechanism(s) by which IL-1β mediates its effect, we measured in the same cultures the levels of IFN-γ and TNF-α, which have been reported to promote autologous tumour-specific cytotoxicity in TIL cultures exogeneously added or endogeneously produced (Wang *et al.*, 1989; Shimizu *et al.*, 1991; Ioannides *et al.*, 1991*c*). As shown in Figure 3, there was a significant increase in IFN-γ levels in MEMNCs cultured with IL-2 and IL-1β (mean value from 20 cultures: $340 \pm 101$ pg ml$^{-1}$) compared with $214 \pm 108$ pg ml$^{-1}$ ($P < 0.01$) produced in the same cultures incubated with IL-2 alone. By comparing the levels of endogeneously produced IFN-γ in MEMNC with IL-2 alone, it becomes clear that these levels were increased in cultures which displayed autologous tumour-specific cytotoxicity (compare Figure 3 with Figure 1), thus confirming their role in promoting CTL activity against autologous tumour cells.

**Table III** Phenotype of MEMNCs in long-term cultures with IL-2

| Patient no. | CD3+ | Percentage of cells CD3+ HLA-DR+ | CD3+ CD25+ | Time point of testing | |
|---|---|---|---|---|---|
| 1 | 97 | 86 | 45 | 35[a] | (380)[b] |
| 2 | 89 | 80 | 52 | 28 | (110) |
| 3 | 79 | 72 | 60 | 42 | (200) |
| 4 | 96 | 87 | 38 | 49 | (350) |
| 5 | 87 | 79 | 55 | 46 | (160) |
| 6 | 98 | 72 | 45 | 56 | (120) |
| 7 | 94 | 61 | 51 | 39 | (90) |
| 8 | 97 | 55 | 49 | 52 | (130) |
| 9 | 92 | 86 | 62 | 56 | (390) |
| 10 | 97 | 90 | 71 | 29 | (100) |
| 11 | 97 | 75 | 45 | 47 | (300) |
| 12 | 95 | 82 | 38 | 25 | (200) |
| 13 | 89 | 67 | 52 | 25 | (270) |
| 14 | 92 | 62 | 46 | 20 | (130) |
| 15 | 92 | 56 | 49 | 59 | (550) |
| 16 | 98 | 70 | 52 | 62 | (320) |
| 17 | 85 | 61 | 60 | 32 | (150) |
| 18 | 91 | 56 | 49 | 27 | (100) |
| 19 | 96 | 47 | 39 | 26 | (150) |
| 20 | 99 | 69 | 46 | 36 | (290) |
| Mean ± s.d. | 93 ± 5 | 71 ± 12 | 50 ± 8 | 39 ± 13 | 224 ± 122 |

[a]Days in culture. [b]Numbers in parenthesis indicate expansion index. Cultures were maintained on a small scale expansion data calculated by extrapolation. MEMNC expansion index calculated as the ratio of the maximum number of lymphocytes in culture to the number of lymphocytes at culture initiation.

In contrast to IFN-γ levels, no significant change in the levels of TNF-α was caused by the co-addition of IL-1β in MEMNC cultures [mean values ± s.d. from 20 cultures $615 \pm 152$ pg ml$^{-1}$ (IL-2) *vs* $620 \pm 117$ pg ml$^{-1}$ (IL-2 + IL-1β) (data not shown)]. Thus, increased endogenous production of IFN-γ seems to represent at least one of the signals through which IL-1β enhances autologous tumour-specific cytotoxicity in MEMNC cultures.

### Discussion

Lymphocytes infiltrating solid tumours (TILs) or malignant effusions (MEMNCs) have been repeatedly demonstrated to efficiently lyse tumour cells *in vitro* upon activation with IL-2. Such cytolytic responses can be specific, mediated solely against autologous tumour cells, or non-specific, directed against a panel of tumour cells (including also autologous ones) and tumour cell lines (Baxevanis & Papamichail, 1994). Thus it remains uncertain if autologous tumour-specific T cells exist in certain tumours and not in others. Based upon the *in vitro* demonstration of autotumour-specific CTLs in the ascitic fluid but not in the tumour specimens of patients with the same type of tumour (Heo *et al.*, 1988; Ioannides *et al.*, 1991*a*), there are indications that it may be possible to generate autologous tumour-specific cytotoxicity under appropriate culture conditions. Therefore, much effort is now being aimed at elucidating the regulatory function of numerous cytokines associated with and potentially involved in the induction of CTLs with specificity for autologous tumour cells among TILs or MEMNCs. The elucidation of regulation of anti-tumour cytolytic responses is important not only for understanding the interaction of the immune system with tumour cells but also for the potential development of effective methods in cancer immunotherapy. Previous studies from our laboratory have shown that exogenous IFN-γ acts synergistically with IL-2 to promote autologous tumour-reactive CTLs among TILs or MEMNCs (Papamichail & Baxevanis, 1992) and to augment anti-tumour cytolytic responses in patients with cancer (Baxevanis *et al.*, 1993*c*). Addition of TNF-α to TILs or MEMNCs cultured in IL-2 leads to preferential outgrowth of CD8+ cells, more

**Figure 3** Levels of IFN-γ measured in the supernatants of MEMNC cultures incubated with IL-2 (**a**) or IL-2+IL-1α (**b**). Supernatants were collected at the time points when phenotype and cytotoxicity were determined. Increases in IFN-γ levels were stastically significant ($P < 0.05$) in all but four cases (patient nos. 2, 10, 16 and 20). Results are means from triplicates. The s.d. was negligible and thus is not shown.

restricted target specificity and up-regulation of IL-2 receptor on the ativated CD8+ cells (Vaccarello *et al.*, 1990; Ioannides *et al.*, 1991*c*). The addition of IFN-γ or TNF-α to TILs cultured in the presence of TNF-α and IL-2 significantly augmented cytotoxicity against autologous tumour (Shimizu *et al.*, 1991).

In the present study we report a synergistic effect of IL-1β with IL-2 for the preferential outgrowth of CD8+ CTLs from lymphocytes infiltrating malignant effusions from patients with metastatic cancer. This expansion resulted in increased levels of cytotoxicity against autologous tumour cells and was associated with increased production of IFN-γ in the MEMNC cultures. IL-1β alone was not capable of inducing expansion or cytotoxicity in MEMNCs (data not shown). To our knowledge our data provide novel evidence for the involvement of IL-1β in the selective increase of autologous tumour-reactive CD8+ CTLs among MEMNCs, since so far synergy of IL-1 and IL-2 has been demonstrated only in the generation of LAK activity (Crump *et al.*, 1989), whereby IL-2 up-regulated the expression of IL-2 receptor on peripheral blood mononuclear cells (PBMCs). Indirect evidence for the involvement of IL-1 in the generation of autologous tumour CTLs was provided in a previous report (Osband *et al.*, 1990), in which high levels of IL-1 in autologous lymphokine mixtures (ALMs) were shown to associate with successful cellular adoptive immunotherapy in patients with renal cell carcinoma using PBMCs activated *in vitro* with ALMs (Osband *et al.*, 1990). Although not yet precisely analysed, IL-1β seems to mediate its effect indirectly by stimulating increased production of IFN-γ. IFN-γ is known to be involved in CTL generation (Chen *et al.*, 1986; Gromo *et al.*, 1987) and activation of CD8+ cells (Siegel, 1988). Moreover, increased production of IFN-γ could be measured in IL-2 + TNF-α-stimulated TIL cultures, which exhibited preferential cytotoxicity for autologous tumour cells (Wang *et al.*, 1989). Thus IFN-γ represents at least one of the signals involved in the IL-1β-driven outgrowth of autologous tumour-reactive CD8+ cells among MEMNCs. The *in vivo* consequences of cellular adoptive immunotherapy using MEMNCs activated *in vitro* by mixtures of IL-2 with IL-1β are as yet unknown. IFN-γ has direct tumoricidal activities and also enhances expression of tumour MHC molecules and tumour-specific antigens, thereby increasing the possibility of immune recognition of tumour cells (Carrel *et al.*, 1985; Stotter *et al.*, 1989). Attempts to treat solid human tumours by the systemic administration of IFN-γ (Garnick *et al.*, 1988; Laszlo *et al.*, 1990) have been mostly unsuccessful (although encouraging results in melanoma and sarcoma patients have been reported; Lienad *et al.*, 1992), possibly because the dose-limiting toxicities induced by IFN-γ precluded delivery of effective cytokine concentrations to tumour sites. Intratumoral production of IFN-γ by the *in vitro*-activated MEMNCs may be above levels that are attainable with systemic administration and thus sufficient to induce anti-tumour effects *in vivo*.

Collectively our data provide new information on the selective outgrowth of autologous tumour-reactive CTLs among MEMNCs. IL-1β has been shown to act synergistically with IL-2 in this respect, with a parallel increase of IFN-γ levels in culture. Further studies are surely required to better analyse the mechanism of action of an IL-2 plus IL-1β combination on the growth, activation and functionality of the autologous tumour-specific CD8+ CTLs. Information obtained from such studies may improve the results of clinical trials in cellular adoptive immunotherapy of cancer.

We wish to thank Miss Joanna Doukoumopoulou for her excellent secretarial assistance.

### References

BAXEVANIS, C.N. & PAPAMICHAIL, M. (1994). Characterization of the anti-tumor immune response in human cancers and strategies for immunotherapy. *Crit. Rev. Oncol. Hematol.*, **16**, 157–179.

BAXEVANIS, C.N., THANOS, D., RECLOS, G.J., ANASTASOPOULOS, E., TSOKOS, G.C., PAPAMATHEAKIS, J. & PAPAMICHAIL, M. (1992). Prothymosin α enhances human and murine MHC class II surface antigen expression and messenger RNA accumulation. *J. Immunol.*, **145**, 1979–1984.

BAXEVANIS, C.N., RECLOS, G.J., GRITZAPIS, A.D., DEDOUSSIS, G.V.Z., ARSENIS, P., KATSIYIANNIS, A., MITSIS, P.G., TSAVARIS, N. & PAPAMICHAIL, M. (1993*a*). Comparison of immune parameters in patients with one or two primary malignant neoplasms. *Natl Immun.*, **12**, 41–49.

BAXEVANIS, C.N., RECLOS, G.J. & PAPAMICHAIL, M. (1993*b*). Prothymosin α restores the depressed allogeneic cell-mediated lympholysis and natural killer cell activity in patients with cancer. *Int. J. Cancer*, **54**, 264–268.

BAXEVANIS, C.N., RECLOS, G.J., GRITZAPIS, A.D., DEDOUSSIS, G.V.Z., MISSITZIS, I. & PAPAMICHAIL, M. (1993*c*). Elevated prostaglandin $E_2$ production by monocytes is responsible for the depressed levels of natural killer cell function in patients with breast cancer. *Cancer*, **72**, 491–501.

BLANCHARD, D.K., KAVANAGH, J.J., SINKOVICS, J.G., CAVANAGH, D., HEWITT, S.M. & DJEU, J.Y. (1988). Infiltration of interleukin-2 inducible killer cells in ascitic fluid and pleural effusions of advanced cancer patients. *Cancer Res.*, **48**, 6321–6327.

CARREL, S., SCHMIDT-KESSEN, A. & GIUFFRE, L. (1985). Recombinant interferon-γ can induce the expression of HLA-D and -DC on DR-negative melanoma cells and enhance the expression of HLA-ABC and tumor-associated antigens. *Eur. J. Immunol.*, **15**, 118–123.

CHEN, L.K., MATHIEU-MAHUL, D., BACH, F.H., DAUSSET, J., BENSUSSAM, A. & SASPORTES, M. (1986). Recombinant interferon α can induce rearrangement of T-cell antigen receptor α-chain genes and maturation to cytotoxicity in T-lymphocyte clones in vitro. *Proc. Natl Acad. Sci. USA*, **83**, 4887-4889.

COZZOLINO, F., TORCIA, M., CAROSSINO, A.-M., GIORDANI, R., SELLI, C., TALINI, G., REALI, E., NOVELLI, A., PISTOLA, V. & FERRARINI, M. (1987). Characterization of cells from invaded lymph nodes in patients with solid tumors. *J. Exp. Med.*, **166**, 303-318.

CRUMP, W.L., OWEN-SCHAUB, L.B. & GRIMM, E.A. (1989). Synergy of human recombinant interleukin-1 with interleukin-2 in the generation of lymphokine-activated killer cells. *Cancer Res.*, **49**, 149-156.

DINNARELLO, C.A., IKEJIMA, T., WARNER, S.J.C., ORENCOLO, S.F., LONNEMANN, G., LANNON, J.G. & LIBB, P. (1987). Interleukin-1 induction of interleukin-1. I. Induction of circulating interleukin-1 in rabbits in vivo and in human mononuclear cells in vitro. *J. Immunol.*, **134**, 3868-3873.

FALKOFF, R.J.M., BUTHER, J.L., DINARELLO, C.A. & FAUCI, A.S. (1984). Direct effects of a monoclonal B cell differentiation factor and a purified interleukin 1 on B cell differentiation. *J. Immunol.*, **133**, 692-697.

FERRINI, S., BIASSONI, R., MORETTA, A., BRUZZONE, M., NICOLIN, A. & MORETTA, L. (1985). Clonal analysis of T lymphocytes isolated from ovarian carcinoma ascitic fluid. *Int. J. Cancer*, **36**, 337-343.

FORNI, G., MUSSO, T., JEMMA, C., BORASCHI, D., TAGLIABUE, A. & GIOVARELLI, M. (1989). Ability of a nonapeptide of the human IL-1β to recruit anti-tumor reactivity in recipient mice. *J. Immunol.*, **142**, 712-718.

FREIMUTH, W.W., DEPPER, J.M. & NABEL, G.J. (1989). Regulation of the IL-2 receptor α-gene interaction of a KB binding protein with cell-specific transcription factors. *J. Immunol.*, **143**, 3064-3070.

GARNICK, M.B., REICH, S.D., MAXWELL, B., CORAL-GOLDSMITH, S., RICHIE, J.P. & RUDNICK, S.A. (1988). Phase I II study of recombinant interferon gamma in advanced renal cell carcinoma. *J. Urol.*, **139**, 251-256.

GROMO, G., GELLER, R.L., INVERARDI, L. & BACH, F.H. (1987). Signal requirements in the step-wise functional maturation of cytotoxic T lymphocytes. *Nature*, **327**, 424-426.

HANNET, I., ERKELLER-YUKSEL, F., LYDYARD, P., DENEYS, V. & DE BRUYERE, M. (1992). Developmental and maturational changes in human blood lymphocytes subpopulations. *Immunol. Today*, **13**, 215-218.

HEO, D.S., WHITESIDE, T.L., KANBOUR, A. & HERBERMAN, R.B. (1988). Lymphocytes infiltrating human ovarian tumors. *J. Immunol.*, **140**, 4022-4049.

IOANNIDES, C.G., PLATSOUCAS, C.D., RASHED, D., WHARTON, J.T., EDWARDS, C.L. & FREEDMAN, R.S. (1991*a*). Tumor cytolysis by lymphocytes infiltrating ovarian malignant ascites. *Cancer Res.*, **51**, 4257-4256.

IOANNIDES, C.G., FREEDMAN, R.S., PLATSOUCAS, C.D., RASHED, S. & KIM, Y.P. (1991*b*). Cytotoxic T cell clones isolated from ovarian tumor-infiltrating lymphocytes recognize multiple antigenic epitopes on autologous tumor cells. *J. Immunol.*, **146**, 1700-1707.

IOANNIDES, C.G., RASHED, S., FISK, B., FAN, D., ITOH, K. & FREEDMAN, R.S. (1991*c*). Lymphocytes infiltrating ovarian malignant ascites: modulation of IL-2-induced proliferation by IL-4 and of selective increase in CD8+ T cells by TNF-α. *Lymphokine Cytokine Res.*, **10**, 307-315.

KAWAKAMI, Y., ROSENBERG, S.A. & LOTZE, M.T. (1988). Interleukin 4 promotes the growth of tumor-infiltrating lymphocytes cytotoxic for human autologous melanoma. *J. Exp. Med.*, **168**, 183-2191.

LASZLO, J., GOLDSTEIN, D., GOCKERMAN, J., HOOD, L., HUANG, A.T., TRIOZZI, P., SEDWICK, W.D., KOREN, H., ELLINWOOD, E.H. & TSO, C.Y. (1990). Phase I studies of recombinant interferon-γ. *J. Biol. Resp. Modif.*, **9**, 185-192.

LIENARD, D., EWALENKO, P., DELMOTTE, J.-J., RENARD, N. & LEJEUNE, F.J. (1992). High dose recombinant tumor necrosis factor alpha in combination with interferon gamma and melphalan in isolation perfusion of the limbs for melanoma and sarcoma. *J. Clin. Oncol.*, **10**, 52-60.

MIZEL, S.B. (1982). Interleukin 1 and T cell activation. *Immunol. Rev.*, **63**, 51-72.

OSBAND, M.E., LAVIN, P.T., BABAYAN, R.K., GRAHAM, S., LAUM, D.L., PARKER, B., SAWCZUK, I., ROSS, S. & KRANE, R.J. (1990). Effect of autolymphocyte therapy on survival and quality life in patients with metastatic renal-cell carcinoma. *Lancet*, **335**, 994-998.

PAPAMICHAIL, M. & BAXEVANIS, C.N. (1992). Gamma-interferon cytotoxic capacity of interleukin-2-induced LAK cells, TIL, and effusion associated lymphocytes. *J. Chemother.*, **4**, 387-393.

PARKINSON, D.R., FISHER, R.I., RAYNER, A.A., PAIETTA, E., MARGOLIN, K.A., WEISS, G.R., MIER, J.W., SZNOL, M., GAYNOR, E.R., BAR, M.H., GUCALP, R. BOLDT, D.H., MILLS, B. & HAWKINS, M.J. (1990). Therapy of renal cell carcinoma with interleukin-2 and lymphokine-activated killer cells. *J. Clin. Oncol.*, **8**, 1620-1626.

PLEBANSKI, M., ELSO, C.J. & BILLINGTON, W.D. (1992). Dependency on interleukin-1 of primary humans in vitro T cell responses to soluble antigens. *Eur. J. Immunol.*, **22**, 2353-2358.

ROSENBERG, S.A., PACKARD, B.S., AEBERSOLD, P.M., SOLOMON, D., TOPALIAN, S.L., TOY, S.T., SIMON, P., LOTZE, M.T., YANG, J.C., SEIPP, C.A., SIMPSON, C., CARTER, C., BOCK, S., SCHWAZENTRUBER, D., WEI, J.P. & WHITE, D.E. (1988). Use of tumor-infiltrating lymphocytes and interleukin-2 in the immunotherapy of patients with metastatic melanoma. *New Engl. J. Med.*, **319**, 1676-1680.

SCHMIDT, J. & TOCCI, M.J. (1990). Interleukin-1. *Handbook Exp. Pharmacol.*, **95**, 1902-1909.

SHIMIZU, Y., IWATSUKI, S., HERBERMAN, R.B. & WHITESIDE, T.L. (1991). Effects of cytokines on in vitro growth of tumor-infiltrating lymphocytes obtained from human primary and metastatic liver tumors. *Cancer Immunol. Immunother.*, **32**, 280-288.

SIEGEL, S.D. (1988). Effects of interferon-γ on the activation of human T lymphocytes. *Cell. Immunol.*, **111**, 461-472.

STOTTER, H., WIEBKE, E.A., TOMITA, S., BELLDEGRUN, A., TOPALIAN, S., ROSENBERG, A. & LOTZE, M.T. (1989). Cytokines after target cell susceptibility to lysis. II. Evaluation of tumor infiltrating lymphocytes. *J. Immunol.*, **142**, 1767-1772.

UHL, J., NEWTON, R.C., GIRI, J.G., SANDLIN, G. & HORUK, S. (1989). Identification of IL-1 receptors on human monocytes. *J. Immunol.*, **142**, 1576-1581.

VACCARELLO, L., WANG, Y.L. & WHITESIDE, T.L. (1990). Sustained outgrowth of autotumor-reactive T lymphocytes from human ovarian carcinomas in the presence of tumor necrosis factor-α and interleukin-2. *Hum. Immunol.*, **28**, 216-277.

WANG, Y.L., LUSHING, S., KANBOUR, A., HERBERMAN, R.B. & WHITESIDE, T.L. (1989). Lymphocytes infiltrating human ovarian tumours: synergy between tumor necrosis factor and interleukin 2 in the generation of $CD8^+$ effectors from tumor-infiltrating lymphocytes. *Cancer Res.*, **49**, S979-S985.